# A Radiomics Nomogram for the Preoperative Prediction of Lymph Node Metastasis in Pancreatic Ductal Adenocarcinoma

**DOI:** 10.3389/fonc.2020.01654

**Published:** 2020-08-27

**Authors:** Jiahao Gao, Fang Han, Yingying Jin, Xiaoshuang Wang, Jiawen Zhang

**Affiliations:** Department of Radiology, Huashan Hospital, Fudan University, Shanghai, China

**Keywords:** pancreatic ductal adenocarcinoma, radiomics, texture analysis, nomogram, contrast-enhanced computed tomography

## Abstract

**Purpose:**

To construct and verify a CT-based multidimensional nomogram for the evaluation of lymph node (LN) status in pancreatic ductal adenocarcinoma (PDAC).

**Materials and Methods:**

We retrospectively assessed data from 172 patients with clinicopathologically confirmed PDAC surgically resected between February 2014 and November 2016. Patients were assigned to either a training cohort (*n* = 121) or a validation cohort (*n* = 51). We acquired radiomics features from the preoperative venous phase (VP) CT images. The maximum relevance–minimum redundancy (mRMR) algorithm and the least absolute shrinkage and selection operator (LASSO) methods were used to select the optimal features. We used multivariable logistic regression to construct a combined radiomics model for visualization in the form of a nomogram. Performance of the nomogram was evaluated by the receiver operating characteristic (ROC) curve approach, calibration testing, and analysis of clinical usefulness.

**Results:**

A Rad score consisting of 10 LN status-related radiomics features was found to be significantly associated with the actual LN status (*P* < 0.01). A nomogram that consisted of Rad scores, CT-reported parenchymal atrophy, and CT-reported LN status performed well in terms of predictive power in the training cohort (area under the curve, 0.92), and this was confirmed in the validation cohort (area under the curve, 0.95). The nomogram also performed well in the calibration test and decision curve analysis, demonstrating its potential clinical value.

**Conclusion:**

A multidimensional radiomics nomogram consisting of Rad scores, CT-reported parenchymal atrophy, and CT-reported LN status may contribute to the non-invasive evaluation of LN status in PDAC patients.

## Introduction

Pancreatic ductal adenocarcinoma (PDAC) is notorious for its occult onset and early metastasis. As one of the several top causes of cancer deaths, the 5-year survival rate of PDAC patients is only 7–8% (1, 2). Early radical surgery is the main treatment modality for patients with PDAC. However, owing to the visceral location of the pancreas and the non-specific symptoms in most early PDAC patients, it is extremely difficult to make an early diagnosis of this disease. This results in limited and suboptimal treatment options for most patients (3). With the intensive efforts to develop neoadjuvant chemotherapy and other new therapeutic methods, there is a growing demand for accurate preoperative staging and personalized tailoring of the therapeutic approach in PDAC. PDAC is well known to be accompanied by the occurrence of lymph node metastasis (LNM), with an LNM rate as high as 59% (4). As an important postoperative prognostic factor, the cancer-positive lymph node (LN) is strongly related to poor prognosis in PDAC patients (5–7). Consequently, there is an urgent need to develop a capability to predict LN status precisely before surgery. Currently, the preoperative status of PDAC patients is mainly evaluated by imaging methods such as CT and MRI. Only relatively poor accuracy can be achieved when evaluating the LN status solely from a morphological perspective (for example, by assessing changes in lymph node size, morphology, and intensity). These approaches are not able to provide effective guidance for clinical treatment and are far from satisfactory predictive factors.

Contrast-enhanced CT (CECT) has long been the preferred imaging modality for preoperative staging of PDAC (8, 9) because it facilitates the assessment of tumor size and vascular involvement. Enlarged lymph nodes as indicated by CECT carry a high positive value for predicting outcome in many malignant tumors, and surgeons can select appropriate LN dissection methods based on the CT report (10–12). For PDAC patients, however, due to the complexity of the peripancreatic structures, it is not easy to define whether LNs are abnormally enlarged. Further complications result from the fact that similar enlarged LNs also appear in local inflammation or secondary biliary obstruction, which can confound the judgment of LN involvement in PDAC. Considering the above factors, CT in fact achieves only a mediocre diagnostic performance for LN metastasis in PDAC, especially regarding its sensitivity (13). MRI and positron emission tomography (PET) have also been considered as potentially useful LNM markers, achieving results similar to those of CT in PDAC patients (14). Recently, the use of endoscopic ultrasound-guided fine needle aspiration (EUS-FNA) has been expanding rapidly for the evaluation of pancreatic masses (15–18). However, EUS-FNA is also affected by many particular complexities, including the investigators’ degree of knowledge of cytopathology, the endosonography technique employed, and the locations and characteristics of the accessed lesions (19, 20).

As an emerging discipline that has attracted numerous researchers’ interests, radiomics extracts multidimensional features contained in available images with high-throughput methods and explores their underlying associations with pathophysiological changes. Recently, several investigators have constructed radiomics models for preoperative LN evaluation in certain gastrointestinal cancers and have succeeded in achieving the desired level of predictive accuracy (21–23). However, to the best of our knowledge, thus far, there are few studies on the development of a radiomics nomogram to predict the LN status for patients with PDAC. To this end, we sought to build and verify a radiomics-based nomogram that could potentially assist in clinical decision-making processes for patients with PDAC.

## Materials and Methods

### Patient Population

Our retrospective study was approved by the Institutional Review Board of Huashan Hospital, with informed consent waived. The PDAC patients who elected to undergo tumor resection and LN dissection between February 2014 and November 2016 in our hospital were retrospectively evaluated. The inclusion criteria were (1) PDAC patients with histological confirmation; (2) thin-layer CECT performed within 1 month before surgery; (3) patients without previous radiotherapy, surgery, and/or chemotherapy; and (4) patients who underwent pancreaticoduodenectomy and where pathologically evidence of LN status was available.

The exclusion criteria were (1) difficulties in distinguishing the tumors on CT images owing to artifacts or for any other reason; (2) features that could not be successfully extracted from the CT images of the patients; and (3) patients with other coexisting primary malignancies. The detailed selection steps for the patients with PDAC are depicted in Supplementary Figure S1.

Ultimately, 172 patients who met the above criteria were included in this retrospective study. Of these, 121 patients were assigned to the training cohort (64 men and 57 women), with an average age of 63.5 ± 9.2 years (range, 35–83). Another 51 patients (30 men, 21 women) with a mean age of 63.7 ± 8.5 years (range, 45–84) constituted the validation cohort.

**FIGURE 1:**
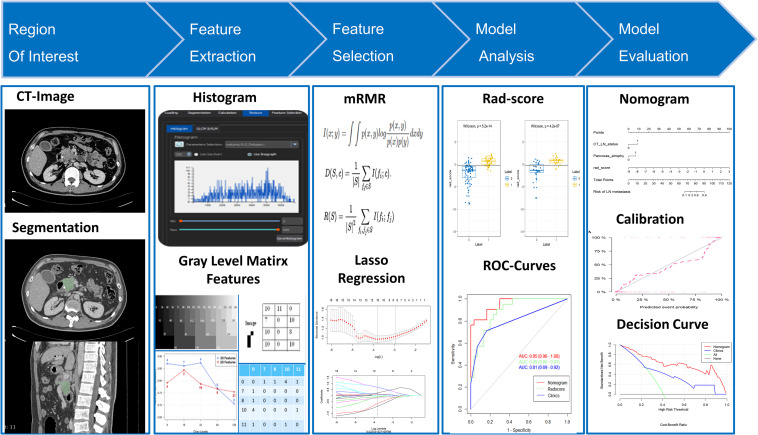
Radiomics workflow.

Clinical data (for example, age, primary tumor site, and preoperative CA-199 level) were obtained from the medical records. Two radiologists (with experience of CT diagnosis of 6 and 10 years, respectively) who knew nothing about the histopathological condition of each patient were appointed to reevaluate the LN status and CT findings (for example, size, periphery, pancreatic duct dilatation, parenchymal atrophy, and vascular invasion). With reference to the relevant literature and clinical diagnostic experience, the CT diagnostic criteria for metastasis to the lymph nodes in PDAC patients were as follows: the peripancreatic and retroperitoneal lymph nodes with short axis diameter > 10 mm, uneven density, uneven enhancement, internal necrosis, blurred edge, and involvement of surrounding organs or vessels (13, 24). If different opinions from the two radiologists were received for the same patient, an independent expert with 22 years of experience in radiological diagnosis was invited to participate in the discussion to decide the final result. A flow diagram of the whole study is depicted in Figure 1.

### Image Acquisition

CT images were acquired from all patients using a 256-slice CT scanner (Brilliance iCT, Philips Medical Systems, Netherlands). The CT scan settings were set as 120 kV; 150–200 mA s; rotation time, 0.75 s; detector collimation, 128 × 0.625 mm; field of view, 350 × 350 mm; matrix, 512 × 512; and slice thickness, 1.5 mm. An anionic contrast medium was injected using an automatic injector at a dose of 1.5 ml/kg at 3.0 ml/s. Arterial phase images were obtained 30 s after contrast medium injection, while venous phase (VP) scans were obtained 45 s after the arterial phase acquisition. All images were uploaded to the picture archiving and communication system (PACS) for further examination.

### Tumor Segmentation and Extraction of Radiomics Features

Feature extraction was carried out on 1.5-mm VP CT images because of their better tumor background contrast (25). The window width and window level applied to the tumor segmentation process were 300 and 40 HU, respectively. One radiologist (HF), with 10 years of experience, manually segmented the tumor on each slice around its edge using open-source image processing software (3D Slicer version 4.11.0; Boston, MA, United States). A total of 396 radiomics features was extracted by the software Artificial Intelligence Kit (GE Healthcare, China). The extracted radiomics features were classified into six categories: Histogram features (*n* = 42), form factor features (*n* = 9), gray level co-occurrence matrix (GLCM) features (*n* = 144), run length matrix (RLM) features (*n* = 180), gray level size zone matrix (GLSZM) features (*n* = 11), and Haralick features (*n* = 10). A detailed description of these features can be seen in Supplementary Material I. We calculated all the features in the segmented tumor region within a three-dimensional volume.

To evaluate the reproducibility and accuracy of the features, two radiologists (HF and GJH) reassessed the tumor segmentation of 60 randomly selected patients after 20 days. The two radiologists were both blinded to the clinical diagnosis and pathological condition of each patient. The inter- and intraclass correlation coefficients (ICCs) were taken as measures of good reproducibility. The threshold of the ICC value for a feature with outstanding reproducibility was deemed to be above 0.75 (26).

### Feature Selection and Signature Construction

For dimensionality reduction and to avoid overfitting, we designed a three-step procedure to select the optimal features. First, we used both intra- and inter-ICC values > 0.75 as a threshold standard to select the stable radiomics features for the next step. Second, an maximum relevance–minimum redundancy (mRMR) method was selected to eliminate the redundant and irrelevant features, such that 30 features were retained for subsequent selection. Finally, we applied the least absolute shrinkage and selection operator (LASSO) regression algorithm to choose the most reproducible and active characteristics from the remaining 30 features. Those features with non-zero coefficients after the cross-validation penalty procedure in the LASSO regression were assigned to construct the Radiomics score (Rad score) in the training cohort, through a linear combination of their weighted coefficients. The relationship between the Rad score and actual LN status was evaluated in both the training cohort and validation cohort by using a Mann–Whitney *U* test. We also used receiver operating characteristic (ROC) testing and area under the curve (AUC) analysis to estimate whether the Rad score could correctly distinguish the actual LN status for PDAC patients in both of the two cohorts.

### Model Building and Nomogram Development

Univariate analyses were performed on all the clinical and conventional imaging features in the training cohort (including age, gender, CA-199 level, tumor size, tumor location, periphery, CT-reported LN status, CT-reported pancreatic atrophy, CT-reported vascular invasion, and CT-reported pancreatic duct dilatation). A multivariable logistic regression with backward stepwise selection was then conducted by using the variables with *P* < 0.1 in the univariable regression. Using the likelihood ratio test with Akaike’s information criterion as the stopping rule, a clinical model was built from those variables with *P* < 0.1 in the multivariate analysis (27, 28). Finally, we constructed a combined multivariable logistic model with Rad scores and the most significant features in the clinical model. To further avoid collinearity, we implemented collinearity diagnosis by checking the variance inflation factor (VIF) for all the factors in the combined model. Those factors with VIF > 5 were excluded from the final model. In order to develop a more understandable evaluation method, we generated a nomogram on the strength of the combined model constructed from the training cohort. Nomogram scores are capable of quantifying the risk of LNM objectively, which can aid in clinical decision-making.

### Model Validation

We compared the discriminatory performance of the established models with the ROC curves and AUC values. Thereafter, we used the calibration curves and Hosmer–Lemeshow test to assess the calibration of the nomogram. The above performance of the model was also verified in the validation cohort. We also performed a stratified analysis of the nomogram to test its evaluation efficiency for different human characteristics (*n* = 172). The adequacy of the performance of the nomogram was assessed by measuring the ROC curves and AUC values in the subgroups including age [≤60 years (young) or >60 years (older)], gender (male or female), and CT-reported LN status (positive or negative).

### Clinical Use

For the purpose of determining the value of our nomogram for clinical applications, we adopted decision curve analysis (DCA) to further compare the net benefit obtained by the deployment of the nomogram and the clinical model. The performance of these two models was evaluated at different threshold probabilities, and the model that possessed larger regions under the curves was selected for the better clinical outcome (29).

### Statistical Analysis

The Student’s *t* test was adopted to compare normally distributed variables. Continuous variables that were not normally distributed were analyzed using the Mann–Whitney *U* test. The discrete variables were compared with the chi-square test. All the statistical analyses that we used in this study were run on R software (version 3.6.2). A detailed description of the R packages that we adopted is provided in Supplementary Material II. A two-tailed *P* < 0.05 was deemed as possessing statistical significance.

## Results

### Patients’ Characteristics

Table 1 summarizes the baseline information of all the patients in this study. There were no significant differences between any of the clinical features of the training and the validation groups, neither for patients with or for those without LN metastasis. Thus, there was a good degree of equivalence between the two groups. Only CT-reported LN status and CT-reported parenchymal atrophy showed a significant difference (*P* < 0.05) between the LNM (+) and LNM (−) group in both the training and the validation cohort.

**TABLE 1:** Characteristics of patients in the training and validation cohorts.

Characteristics	Training cohort (*n* = 121)			Validation cohort (*n* = 51)		
		
	LN Metastasis(+)	LN Metastasis(−)	*P*	LN Metastasis(+)	LN Metastasis(−)	*P*
Age, mean ± SD	64.1 ± 8.6	62.2 ± 9.6	0.260	64.8 ± 10.3	62.2 ± 9.4	0.364
Sex, No(%)						
Male	42 (58.3)	22 (44.9)	0.205	19 (63.3)	11 (52.4)	0.622
Female	30 (41.7)	27 (55.1)		11 (36.7)	10 (47.6)	
CA-199 level, No(%)						
Normal	11 (15.3)	11 (22.4)	0.444	12 (40.0)	2 (9.5)	0.037
Abnormal	61 (84.7)	38 (77.6)		18 (60.0)	19 (90.5)	
Tumor size on CT (cm)	3.3 ± 1.4	3.7 ± 1.7	0.267	3.4 ± 1.3	4.1 ± 2.1	0.141
Primary site						
Head and neck	40 (55.6)	31 (63.3)	0.511	14 (46.7)	14 (66.7)	0.260
Body and tail	32 (44.4)	18 (36.7)		16 (53.3)	7 (3.3)	
Margin						
Well-defined	7 (9.7)	3 (6.1)	0.711	0 (0.0)	2 (9.5)	0.321
Poorly defined	65 (90.3)	46 (93.9)		30 (100.0)	19 (90.5)	
Parenchymal atrophy						
Yes	5 (6.9)	12 (24.5)	0.014	3 (10.0)	8 (36.1)	0.040
No	67 (93.1)	37 (75.5)		27 (90.0)	13 (61.9)	
Pancreatic duct dilatation						
Yes	33 (45.8)	32 (65.3)	0.054	14 (46.7)	9 (42.9)	0.992
No	39 (54.2)	17 (34.7)		16 (53.3)	12 (57.1)	
CT-reported T stage						
T1	17 (23.6)	9 (18.4)	0.853	5 (16.7)	2 (9.5)	0.296
T2	35 (48.6)	24 (49.0)		19 (63.3)	10 (47.6)	
T3	15 (20.8)	11 (22.4)		5 (16.7)	6 (28.6)	
T4	5 (6.9)	5 (10.2)		1 (3.3)	3 (14.3)	
CT-reported vascular invasion						
Yes	7 (9.7)	7 (14.3)	0.631	2 (6.7)	6 (28.6)	0.084
No	67 (93.1)	42 (85.3)		28 (93.3)	15 (71.4)	
CT-reported LN status						
LN-negative	62 (86.1)	21 (42.9)	<0.001	28 (93.3)	9 (42.9)	<0.001
LN-positive	10 (13.9)	28 (57.1)		2 (6.7)	12 (57.1)	
Radiomics score, median (interquartile range)	−1.3(−2.9,−0.5)	0.7(0.0,1.3)	<0.001	−1.3(−1.8,−0.1)	0.8(0.3,1.2)	<0.001

### Feature Selection and Radiomics Signature Construction

**FIGURE 2:**
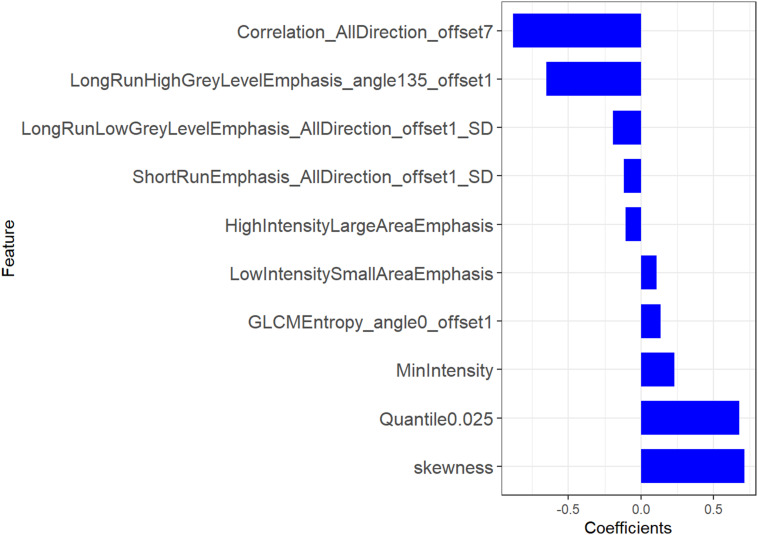
Radiomic features selected for signature building.

A total of 396 features were extracted from axial VP CE-CT scans, of which 335 (81.9%) radiomics features were retained after the ICC assessment. Of these, 30 features were retained through the mRMR algorithm for the subsequent LASSO analysis. The LASSO regression was conducted to select the optimized features to construct the final model. Thus, finally, 10 radiomics features were chosen to build the radiomics signature. A detailed description of the selected features can be seen in Figure 2 and Supplementary Table S1. A multilogistic regression-based radiomics signature was constructed using these 10 features, which are represented by the quantitative index designated the Rad score. The formula for calculating the Rad score is presented in Supplementary Material III.

### Evaluation of the Performance of the Rad Score

**FIGURE 3:**
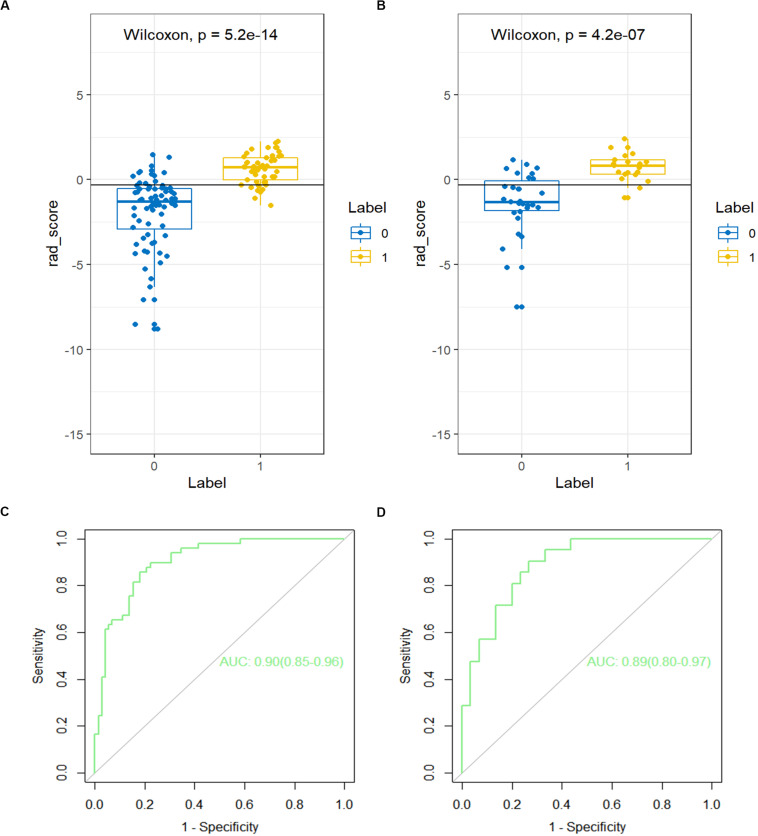
The receiver operating characteristic (ROC) curves of the Rad score in the **(A)** training cohort and the **(B)** validation cohort. The box-dot plots of the Rad scores in the **(C)** training cohort and the **(D)** validation cohort. The orange markers indicate patients with LNM; the green markers indicate patients with non-LNM. The black horizontal line presents the threshold. Patients with Rad scores higher than −0.2635 are classified as LNM; patients with scores lower than −0.2635 are classified as non-LNM.

A significant difference can be seen in the Rad score between patients with or without LN metastasis in the training cohort (*P* < 0.01), which is confirmed in the validation cohort (*P* < 0.01; Figure 3A). The Rad score presented an AUC value of 0.90 [95% confidence interval (CI), 0.85, 0.96] in the training cohort and 0.89 (95% CI, 0.80, 0.97) in the validation cohort, documenting very good discriminatory abilities (Figure 3B).

### Nomogram Development and Performance Validation

**FIGURE 4:**
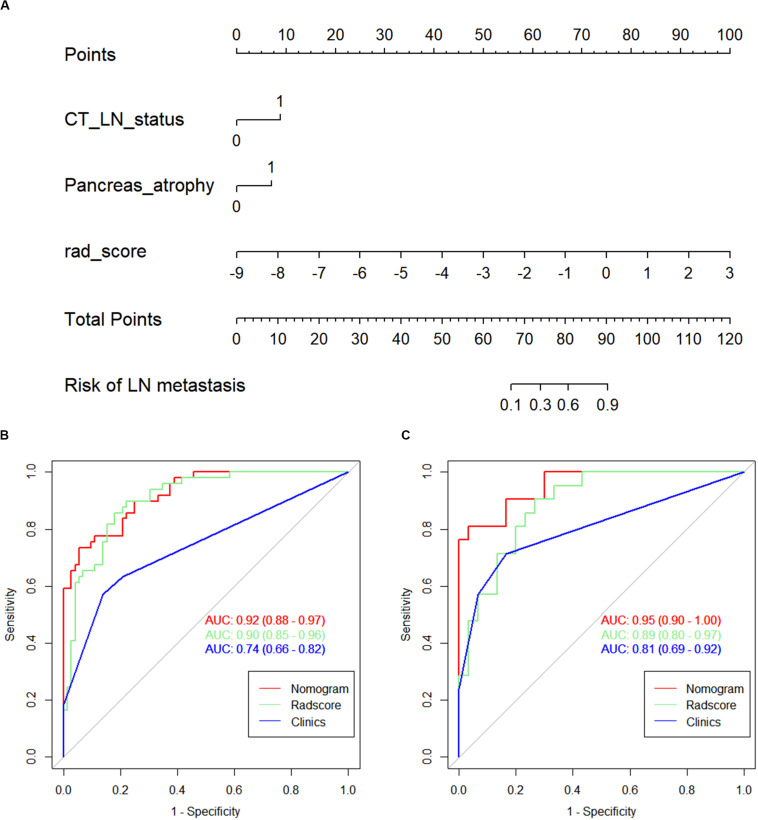
**(A)** The nomogram, combining Rad score, CT-reported parenchymal atrophy, and CT-reported LN status. Receiver operating characteristic (ROC) curves for the nomogram, Rad score, and clinical model in the **(B)** training and **(C)** validation cohorts.

A detailed description of the multivariable regression analysis can be seen in Table 2. Rad scores, CT-reported LN status, and CT-reported parenchymal atrophy were all significantly correlated with LNM. We constructed a combined model that incorporated Rad scores and the two conventional imaging features and established a nomogram based on this combined model (Figure 4A). In the ROC test, the nomogram displayed a superb ability for evaluating LNM in PDAC patients, with AUCs of 0.92 (95% CI, 0.88–0.97) and 0.95 (95% CI, 0.90–1.00) in the training and validation cohorts, respectively (Figures 4B,C and Table 3). The application of Delong’s test showed that significant differences are present in the AUC values between the combined nomogram and the clinical model (*P* < 0.001), which confirm its satisfactory predictive performance.

**TABLE 2:** Risk factors for lymph node metastasis in PDAC.

Intercept and variable	Combined model (95% CI)		Clinical Model (95% CI)	
		
	Odds ratio	*P*	Odds ratio	*P*
Intercept	0.52 (0.26,1.02)	<0.01	0.29 (0.18,0.49)	<0.01
Parenchymal atrophy	3.69 (0.75,21.07)	0.09	3.47 (1.04,12.78)	0.05
Pancreatic duct dilatation	NA	NA	0.37 (−0.50,1.24)	0.40
CT-reported LN status	5.23 (1.59,19.25)	<0.01	7.63 (3.22,19.36)	<0.01
Rad score	4.75 (2.68,9.88)	<0.01	NA	NA

*NA, not available.*

**TABLE 3:** Diagnostic performance of models in the training and validation cohorts.

Models	Training cohort (*n* = 121)				Validation cohort (*n* = 51)			
		
	Sensitivity	Specificity	Accuracy (95% CI)	AUC (95% CI)	Sensitivity	Specificity	Accuracy (95%CI)	AUC (95% CI)
Clinical model	0.57	0.86	0.74 (0.66,0.82)	0.74 (0.66,0.82)	0.57	0.93	0.78 (0.65,0.89)	0.81 (0.69–0.92)
Rad-score	0.85	0.81	0.83 (0.76,0.90)	0.90 (0.85–0.96)	0.90	0.73	0.80 (0.67,0.90)	0.89 (0.80–0.97)
Combined nomogram	0.73	0.94	0.86 (0.78,0.92)	0.92 (0.88–0.97)	0.81	0.87	0.84 (0.71,0.93)	0.95 (0.90–1.00)

**FIGURE 5:**
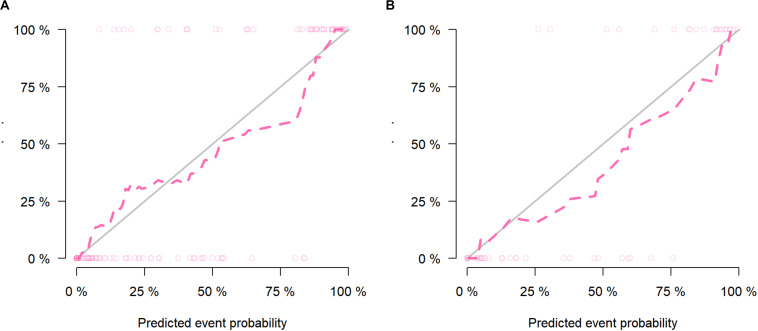
The calibration curves presented good consistency between the nomogram-predicted lymph node (LN) status and observed LN status in the **(A)** training cohort and **(B)** validation cohort.

The calibration curves of the nomogram presented a good consistency between predicted and observed LN status in both training and validation cohorts (Figures 5A,B). The Hosmer–Lemeshow test yielded non-significant *P* values for differences between the two datasets (training cohort, *P* = 0.31; validation cohort, *P* = 0.68), documenting that the goodness of fit of our nomogram was acceptable.

Stratification analysis revealed that the nomogram had a good capacity to identify lymph nodes in different stratification contexts (Table 4). The AUC value for the combined nomogram was 0.88 (95% CI, 0.82, and 0.94) in the CT-reported LN-negative subgroup, demonstrating its improved recognition capability compared with the traditional imaging methods.

**TABLE 4:** The area under the curve (AUC) values of combined model for stratified analysis in different subgroup.

Combined nomogram		Age subgroup		Sex subgroup		CT-reported LN status subgroup	
			
	All group (*n* = 172)	Young (*n* = 65)	Old (*n* = 107)	Male (*n* = 94)	Female (*n* = 78)	CT-LN(+) (*n* = 52)	CT-LN(−) (*n* = 120)
Patients	LNM (+) = 102	LNM (+) = 27	LNM (+) = 43	LNM (+) = 33	LNM (+) = 37	LNM (+) = 40	LNM (+) = 30
	LNM (−) = 70	LNM (−) = 38	LNM (−) = 64	LNM (−) = 61	LNM (−) = 41	LNM (−) = 12	LNM (−) = 90
AUC values (95% CI)		0.965 (0.926,1.000)	0.912 (0.861,0.963)	0.940 (0.895,0.985)	0.918 (0.859,0.976)	0.973 (0.934,1.000)	0.878 (0.816,0.940)

### Clinical Use

**FIGURE 6:**
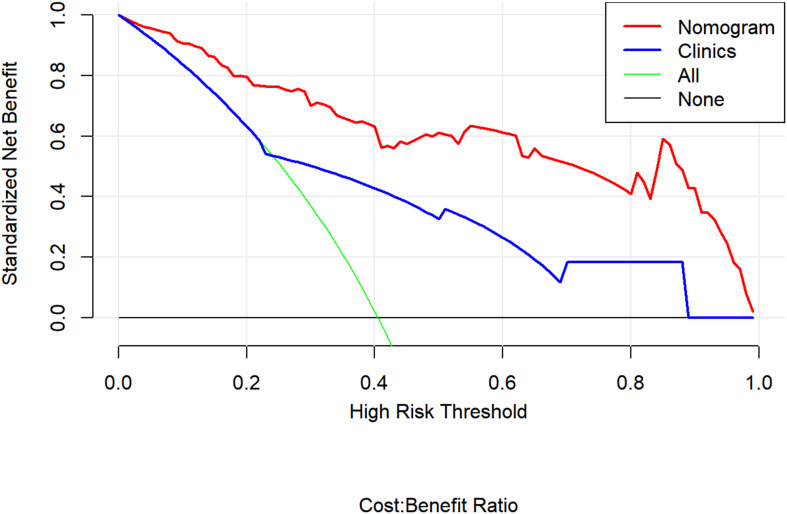
Decision curve analysis for the combined model (nomograms) compared with clinical model in the validation cohort. The decision curve analysis demonstrated that when the threshold probability is over 10% approximately, the nomogram would provide extra diagnostic efficacy over the “treat-all” or “treat-none” scheme and the clinical model.

Figure 6 presents a DCA using our nomogram. It can be concluded from inspecting the curve that when the threshold probability is over 10% approximately, the nomogram would provide extra diagnostic efficacy over and above the “treat all” or “treat none” scheme of the clinical model.

## Discussion

In the present study, we constructed and validated a CT-based radiomics nomogram consisting of the Rad score together with clinical features, which can be used for predicting LN status in PDAC patients. In the ROC test, the combined model and its nomogram exhibited excellent resolution capability in both the training and validation cohorts. The AUC values of the combined model (0.92) and radiomics model (0.90) were both significantly higher than that of the clinical model (0.74). The DCA test showed that the nomogram could effectively facilitate clinical decision-making as well. Considering that only a minority of patients with PDAC can potentially undergo radical resection, an accurate choice of clinical treatment is crucial for PDAC patients. The prognostic value of LN status in PDAC patients has been demonstrated by many investigations, and it is known that even the number of positive LNs also affects therapeutic efficacy (30–32).

Current surgical decision-making depends heavily on imaging diagnosis, despite the fact that the performance of the imaging methodology is not yet completely satisfactory. Although the macroscopic features we observed in the images do have prognostic value for PDAC patients (33), they are not sufficient when it comes to the assessment of LN status. CT is the most often preferred method for preoperative cancer evaluation. In many studies, LNs bigger than 10 mm have been classified as positive LNs (34–36). Nevertheless, the diagnostic efficiency of CT yields only low accuracy and sensitivity. EUS-FNA seems to be a superior solution currently for determining LN metastasis and can be used to extract a piece of tissue and obtain the pathological information on a specific lymph node (37). However, it remains a challenge in clinical practice routinely using endoscopic ultrasonography to access this type of biopsy. Many factors also affect the accuracy of EUS examination, quite randomly (38, 39), thus reducing its value for clinical LN status prediction.

Radiomics detects the heterogeneity of the tumor through the spatial distribution of voxel intensities, acquiring in-depth information from images of the lesion. We constructed a radiomics nomogram to predict LN metastasis by combining the radiomics features and the most relevant clinical characteristics. To facilitate the clinical use of a radiomics model, we constructed a nomogram to visualize and quantize the results of the complex radiomics analysis. Considering the weaknesses of the preceding radiomics models and the doubts about their reproducibility and robustness (40), we took effective measures to guarantee the objectivity and reproducibility of our radiomics model. Changes in tissues that were less correlated with tumor heterogeneity (such as cystic changes) were excluded from the ROI. We preprocessed all the radiomics features to avoid the effects of scale differences. Two radiologists carried out the tumor segmentation step, and ICC coefficients were used to minimize subjectivity and operator error. Both the segmentation and feature extraction software that we used were commonly adopted in earlier investigations and had been verified by those studies (41–43). A three-step approach was devised to reduce the number of features, prevent over fitting, and minimize the collinear features. With all the above measures, a relatively evidence-based and independent radiomics model was constructed for the evaluation of LN status in PDAC patients.

In our radiomics model, we extracted 10 features that could better reflect intratumor heterogeneity and subtle changes in the lesions. The CT-reported LN status and CT-reported parenchymal atrophy also served as independent predictors in the combined model. Previous studies had demonstrated that the CT-reported LN status was significantly related to the pathological LN status in other malignant tumors (44–46), and our study also supports this notion. Fibrosis and parenchymal atrophy is consistently found in PDAC (47), and the tumor microenvironment is likely to be influenced by the reciprocal interactions among fibroblasts and tumor cells in the fibrotic lesions. The degree of pancreatic atrophy is directly related to the malignancy of the tumor and reflects the severity of tissue fibrosis (48). Although to the best of our knowledge, there are no published studies indicating that pancreatic atrophy is an independent factor for LN status in PDAC, we have reason to believe that it does have a potential association with LNM in such patients. Compared with the previous studies using radiomics to evaluate the LN status for PDAC patients (49, 50), we believe that our approach offers advantages for the following reasons: (1) We performed a stratified analysis to further evaluate the prediction efficiency of our model, which can determine the clinical application potential of our model under different conditions. (2) In addition to the CT-reported LN status, we adopted more conventional CT imaging signs in the clinical model. The combination of radiomics and traditional imaging signs may improve the clinical acceptability of radiomics.

Our study has several limitations as follows: (1) One of the main drawbacks of radiomics research is that the poor interpretation of the radiomics features has always hindered the clinical promotion of radiomics. Although many studies tried to generate the correlations from the perspective of grayscale intensity and matrix uniformity, it is still difficult to directly connect the radiomics features with the clinical status. This problem also existed in our study. (2) The patients in our study were all recruited from one hospital. Further external validation with considerably larger data sets should be carried out to testify to the robustness and prediction accuracy of the model. (3) The study only concentrated on the occurrence or lack of LN metastasis in PDAC patients. The number of different metastatic LNs is also important according to the latest cancer staging guidelines (51). The predictive accuracy of radiomics for specific N stage (N1–N2) needs further investigation. (4) Other clinical and imaging features may also be valuable for the construction of the predictive model, but we excluded them from the present study for reasons of data integrity and only selected the most reasonable features.

In conclusion, we have established and verified a novel radiomics nomogram to evaluate LN status in PDAC patients. The model consisted of Rad scores, CT-reported LN status, and CT-reported parenchymal atrophy. Our results demonstrate that the nomogram could likely be conducive to enhancing an accurate auxiliary diagnosis and increasing the optimization of appropriate clinical treatment.

## Data Availability Statement

The datasets generated for this study are available on request to the corresponding author.

## Ethics Statement

The studies involving human participants were reviewed and approved by the Institutional Review Board of Huashan Hospital. Written informed consent for participation was not required for this study in accordance with the national legislation and the institutional requirements.

## Author Contributions

JG and FH contributed equally to the study and designed and carried out the experiments. YJ and XW collected and sorted the data. JZ supervised and revised the manuscript. All authors contributed to the article and approved the submitted version.

## Conflict of Interest

The authors declare that the research was conducted in the absence of any commercial or financial relationships that could be construed as a potential conflict of interest.
