# Supplementary material for: A Radiomics Nomogram for the Preoperative Prediction of Lymph Node Metastasis in Pancreatic Ductal Adenocarcinoma
Source: Front Oncol. 2020 Aug 27;10:1654. doi: 10.3389/fonc.2020.01654 (PMC7482654; doi:10.3389/fonc.2020.01654)
Supplement: Supplementary file 1 [file Data_Sheet_1.docx]

***Supplementary Material***

**A** **Radiomics Nomogram for the preoperative prediction of lymph node metastasis in** **Pancreatic Ductal Adenocarcinoma**

**Authors**

**I. Radiomic features extraction methodology and radiomic signatures.**

**II. R packages we used in the whole process**

**III. Calculation formulas for radiomics model and combination nomogram**

**Table S1. The classification and calculation formula of 10 selected texture features**

**Figure S1. Recruitment pathway for patients in this study**

**I. R****adiomic** **features extraction methodology and radiomic signatures.**

We extracted 396 radiomics features from the axial venous phase CE-CT scans. The characteristics can be divided into six categories: Histogram features(n=42), Form factor features(n=9),Grey Level Co-occurrence Matrix (GLCM) features(n=144), Run Length Matrix (RLM) features(n=180), Grey Level Size Zone Matrix (GLSZM) features(n=11), and Haralick features(n=10). The figure below shows the specific contents of each type of feature. Then, we used the Minimum redundancy maximum relevance algorithm to select the features with much relevance to the response variable. 30 characteristics that are most correlated with the results and least correlated with each other were chosen for the oncoming LASSO regression. Finally, we selected 10 radiomic signatures by the LASSO logistic regression model. Radiomics score was calculated by the formula in Supplementary Material III.


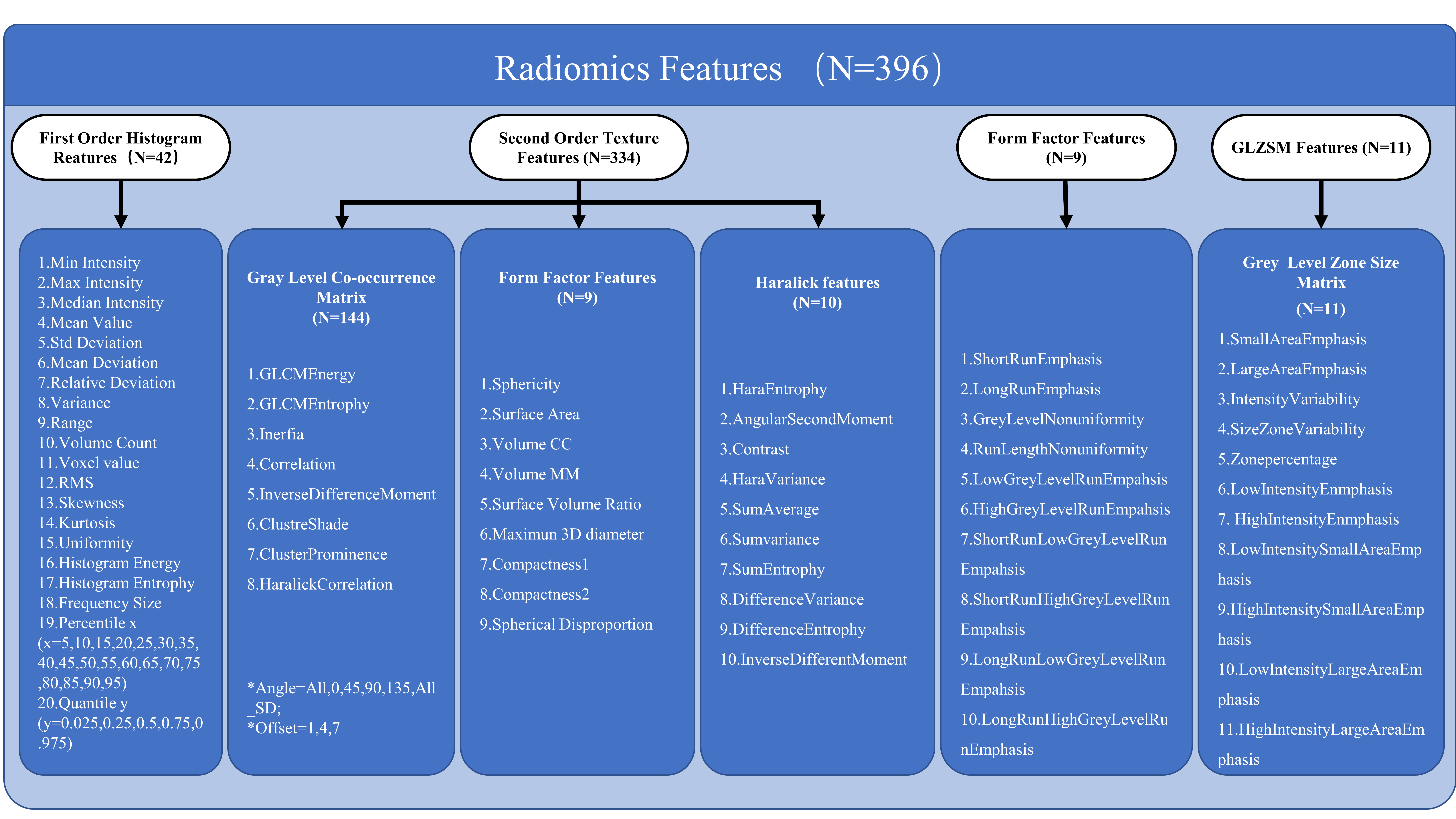


**II.** **R packages we used in the whole process**

The “glmnet” package was used for LASSO logistic regression. The “rms” package was used in the logistic regression analysis , calibration plots and VIF calculation. Calibration curves were constructed using bootstrapping validation with 1,000 resamples. ROC curves and AUC value were calculated using the “pROC” package. The Hosmer-Lemeshow test was performed using the “generalhoslem” package. The “dca.R” package was used for decision curve analysis.

**III.** **Calculation formulas for radiomics model and combination nomogram**

*Rad-score*=

0.232*MinIntensity+0.106*LowIntensitySmallAreaEmphasis-0.116*ShortRunEmphasis_AllDirection_offset1_SD+0.713*skewness-0.192*LongRunLowGreyLevelEmphasis_AllDirection_offset1_SD-0.882*Correlation_AllDirection_offset7-0.653*LongRunHighGreyLevelEmphasis_angle135_offset1-0.107*HighIntensityLargeAreaEmphasis+0.135*GLCMEntropy_angle0_offset1+0.677*Quantile0.025-0.864

*Nomoscore* = -0.649+1.654*CT_LN_status+1.305*Pancreas_atrophy+1.559*rad_score

**Table S1.** **The classification and calculation formula of 10 selected texture features**

| **Category** | **Feature** | **Formula** |
| --- | --- | --- |
| Histogram  GLCM | Skewness  Quantile 0.025  MinIntensity | $\frac{\frac{1}{N}\sum_{i=1}^{N} {(x\left( i \right)-\bar{x})}^{3}}{\left( \sqrt{\frac{1}{n}}\sum_{i=1}^{N} {(x\left( i \right)-\bar{x})}^{2} \right)^{3}}$  I p = N k/q  The minimum intensity value of X  $-\sum_{i,j} \frac{(i-\mu)(j-\mu)g(i,j)}{\sigma^{2}}$  $-\sum_{i,j} g(i,j){log}_{2}(i,j)$  *g is a GLCM  Where i, j are the spatial coordinates of g (i,j). |
|  | Correlation_AllDirection_offset7  GLCMEntropy_angle0_offset1 |  |
| RLM  GLZSM | LongRunHighGreyLevelEmphasis_angle135_offset1  LongRunLowGreyLevelEmphasis_AllDirection_offset1_SD  ShortRunEmphasis_AllDirection_offset1_SD | $\frac{1}{n}_{r}\sum_{\text{j}=i}^{N} \sum_{i=1}^{M} p(i,j,\theta)i^{2}j^{2}$ $\frac{1}{n_{r}}\sum_{j=i}^{N} \sum_{i=1}^{M} \frac{p(i,j,\theta)j^{2}}{i^{2}}$      $\sum_{i} \sum_{j} i^{2}j^{2}p(i,j)$  $\sum_{i} \sum_{j} \frac{p(i,j)}{i^{2}j^{2}}$ |
|  | LowIntensitySmallAreaEmphasis  HighIntensityLargeAreaEmphasis |  |

**Figure S1. Recruitment pathway for patients in this study**

227 patients with PDAC were collected from February, 2014 to November, 2016 met the inclusion criteria

Finally,172 patients were enrolled in this study

15 patients with other coexisting primary malignancies

37 patients excluded with blurring lesions due to artifacts or for any other reason

3 patients whose radiomics features could not be successfully extracted from their CT images.
